# Emotion regulation strategies and mental wellbeing among Chinese college students during COVID-19: the moderating roles of confinement and attentional bias

**DOI:** 10.3389/fpsyg.2025.1571275

**Published:** 2025-06-13

**Authors:** Mengqi Xiao, Yiran Wang, Yaling Zhuang, Yier Luo, Li Liu, Yingxin Lin, Dingguo Gao, Jiahua Xu

**Affiliations:** ^1^Department of Psychology, Sun Yat-sen University, Guangzhou, China; ^2^School of Educational Science, Guangdong Polytechnic Normal University, Guangzhou, China; ^3^Department of Psychology, Guangdong Provincial Key Laboratory of Social Cognitive Neuroscience and Mental Well-being, Guangzhou, China; ^4^Guangdong Provincial Key Laboratory of Brain Function, Guangzhou, China; ^5^Psychiatry Research Center, Beijing Huilongguan Hospital, Peking University Huilonguan Clinical Medical School, Beijing, China

**Keywords:** confinement situation, emotion regulation strategies, mental wellbeing, attentional bias, COVID-19

## Abstract

**Background:**

During COVID-19, confinement measures were implemented to curb the epidemic spread. While effective in reducing infections, these measures likely deteriorated the psychological wellbeing of students due to school closures and isolation.

**Methods:**

This study analyzed 13,109 valid questionnaires from individuals aged 18–24 years (mean 20.28 ± 1.518) to explore how emotion regulation strategies (cognitive reappraisal and expression suppression) impact mental wellbeing through attentional biases (positive and negative), with confinement as three moderators.

**Results:**

Cognitive reappraisal was positively associated with mental wellbeing, whereas expression suppression showed a negative association. Positive attentional bias was associated with higher wellbeing, whereas negative bias was linked to lower levels of it. Negative attentional bias was linked to a stronger positive association between cognitive reappraisal and mental wellbeing, as well as a more pronounced negative association? with expression suppression. Confinement was associated with a stronger positive relation between cognitive reappraisal and mental wellbeing, while corresponding to a more negative relation with expression suppression.

**Conclusion:**

Our findings highlight the complex interplay between emotion regulation strategies and mental wellbeing during confinement. Cognitive reappraisal and positive attentional bias are associated with protective effects, while expression suppression and negative bias are linked to detrimental effects. Confinement measures, despite their positive impact on physical health, significantly modulate these effects. Tailored interventions considering individual differences and contexts are needed to support mental wellbeing in similar crises.

## Introduction

COVID-19, marked by its rapid transmissibility and widespread reach, coupled with a notable fatality rate, imposed a formidable public health challenge. In the face of this ongoing threat, individuals have increasingly grappled with a deluge of negative emotions, including anxiety and fear, which, amidst uncertainty, pose a grave risk to mental wellbeing. Empirical evidence underscores a plethora of exacerbated mental wellbeing symptoms, notably depression, anxiety, and stress, that have surfaced during the pandemic (Cao et al., 2020; Fullana et al., 2020). College students, as a special and crucial group in society, are in a critical stage of psychological development. They highly rely on social interaction and campus environment to achieve self-growth and development (Chavira et al., 2022; Liu et al., 2024; Yang et al., 2021). During pandemic prevention and control, measures such as campus closure, interruption of offline teaching, and social confinement disrupted their regular study and life rhythms, becoming associated with a series of psychological problems (Lansford et al., 2023; Wang et al., 2022; C. Zhang et al., 2020).

### Mental health of college students in the pandemic

During the pandemic, people's mental health was affected to varying degrees (Davico et al., 2024; Özlü-Erkilic et al., 2021). Martin et al. (2025) conducted a systematic review of 36 studies from multiple countries (involving Africa, Asia, Europe, South America, and North America) between 2020 and 2022 to explore the impact of adult self-confinement on mental health. They found inconsistent effects of self-confinement on mental health, but qualitative research showed negative impacts, with individuals with pre-existing physical and mental illnesses and those lacking support being more vulnerable (Martin et al., 2025). Pietrzak and Hanke (2024) conducted a literature review integrating 35 studies and found that mental health problems were more prevalent among young people and women during the pandemic, while changes in suicide attempts showed different trends across countries Pietrzak and Hanke (2024). For example, Kauhanen et al. (2023) conducted a systematic review of 21 articles from 11 countries including China, the UK, and the US, covering 2009–2020, and found that most studies showed a longitudinal deterioration in the mental health of adolescents and young people during the pandemic, including increased symptoms of depression and anxiety (Kauhanen et al., 2023). Hall et al. (2023) found that during the 2022 lockdown in Shanghai, China, the prevalence of depression, anxiety, and suicidal ideation among residents significantly increased, especially the anxiety symptoms in the 18–29-year-old youth group, highlighting the vulnerability of young adults such as college students.

A study narratively reviewed the psychological impact of the pandemic on global children and adolescents and found that the pandemic led to a decline in their mental health (Boonroungrut et al., 2022). Lopez-Serrano et al. (2023) showed that pandemic lockdowns significantly increased externalizing symptoms and behaviors in Spanish children and adolescents (especially those with autism spectrum disorder and conduct disorder), which corroborates the findings of Rider et al. (2021). In a cross-regional comparison within the same country, Kurz et al. (2022) studied different regions in Germany and found that among first-grade primary school students during the pandemic, girls experienced a decline in health-related quality of life and increased emotional and behavioral difficulties, while boys significantly increased their screen time. Salmi et al. (2022) analyzed chat data from the Dutch suicide prevention hotline and found significant differences in suicide-related issues among callers of different genders, ages, and residential statuses during the pandemic, which is consistent with the conclusions of Chadi et al. (2022) and Cooper et al. (2021). Cross-national studies have specifically identified adolescents and young adults as a vulnerable subgroup, whose developmental stage—marked by high social dependency and identity formation—amplifies their susceptibility to pandemic-related disruptions (Lansford et al., 2023; Skinner et al., 2023a,b). For example, a nine-country investigation (Skinner et al., 2022) revealed that young adults who reported higher pre-pandemic positivity and future orientation exhibited significantly weaker associations between COVID-19-related disruption and perceived increases in internalizing symptoms, such as anxiety and depression, underscoring the protective role of cognitive-affective resources.

### Mental health of college students under confinement

Confinement measures, implemented as a crucial tool in disease prevention and control, emerged as an additional predictive factor for psychological deterioration (Rajkumar et al., 2022). Schools, as congregational hubs, were temporarily shuttered to mitigate the spread of COVID-19 and safeguard students from infection. However, this measure inadvertently exposed confined populations, particularly those within academic settings, to heightened vulnerability and predisposition to anxiety, sorrow, and other detrimental emotional states, as corroborated by recent studies (Tepeli Temiz and Elsharnouby, 2022). People in COVID-19 lockdown often experience insomnia (Yang et al., 2023) and exhibit signs of psychiatric subclinical manifestations, characterized by compromised acute stress coping abilities, manifesting as depressive and anxious symptoms (Moreno et al., 2020; Sprang and Silman, 2013). Notably, age demographics such as young adults and the elderly have been disproportionately affected by these adverse psychological sequelae (Amanzio et al., 2023; Muñoz-Navarro et al., 2021), emphasizing the urgency for tailored interventions to address these specific mental health challenges.

The dual effects of quarantine measures arise from their inherently contradictory roles. While confinement measures reduced infection risks, prolonged academic disruptions (e.g., campus closures, online learning challenges) and social isolation amplified uncertainty, particularly among students dependent on structured educational environments (Tasso et al., 2021). Liu et al. (2024) found Chinese college freshmen faced dual challenges of identity transition and university adaptation during prolonged lockdowns, with anxiety and poor sleep significantly affecting mental health. Wu et al. (2025) reported a 28.0% prevalence of post-traumatic stress symptoms among Chinese students, highlighting the pandemic's severe mental health impact. Patrono et al. (2024) linked COVID-19 fear to poor sleep and emotional instability in Chilean students during lockdowns, reflecting youth psychological adaptation challenges.

### Role of confinement

Although existing research has yielded fruitful results, there are still certain limitations. On the one hand, research results are significantly affected by regional differences, including economic development levels, social and political environments, population characteristics, resident mobility, medical resources, and COVID-19 transmission situations (Kwilinski et al., 2022; Rule et al., 2025), while differences in restriction measures implemented by different countries and regions also interfere with research results (World Health Statistics, 2020). In general, the strictness of lockdown measures varies across countries, leading to inconsistent mental health research results (Davico et al., 2024; Nash, 2023; Özlü-Erkilic et al., 2021). On the other hand, epidemiological surveys minimize regional differences through sampling, but to some extent, they overlook potential impacts such as residential density and medical standards.

This study conducts a cross-sectional analysis of two universities in the same city with differing management policies, using a homogeneous regional setting to precisely examine the relationship between college students' emotion regulation strategies and mental health under campus confinement (Solmi et al., 2025). Narrowing the scope to within-city university types reduces interference from regional variables like social culture and economic levels, making findings more targeted compared to broader studies (Jackson et al., 2021). For instance, cross-country or large-region research often faces confounding effects from significant social, cultural, or developmental disparities (Davico et al., 2024; Özlü-Erkilic et al., 2021). By focusing on intra-city universities, this study controls such macro-factors to accurately explore links between confinement measures, emotion regulation, and mental health.

Choosing Chinese college students as the research population holds significant value. In their critical transition from adolescence to adulthood, they face academic, social, and career planning pressures, with sensitive and unstable mental states highly susceptible to external environmental changes (Chavira et al., 2022; Pandya and Lodha, 2022). The pandemic's economic downturn has amplified their employment stress, while the outbreak disrupted their life and study routines, obstructed goals, and increased psychological burdens (Liu et al., 2024; Zarowski et al., 2024). Chinese university residences, characterized by high-density centralized living (Zhang et al., 2022), elevate pandemic transmission risks and cluster outbreak likelihood. Strict campus confinement measures under unified management—implemented to ensure safety—significantly impact students' emotional regulation and mental health (Chavira et al., 2022; Gao et al., 2020; Ocampo et al., 2024).

This special residential environment and confinement methods provide a unique context for the study, helping to deeply explore college students' emotion regulation strategies and their impact on mental health under strict control. Research on Chinese college students under pandemic confinement measures can not only provide a scientific basis for Chinese universities to develop targeted mental health interventions but also offer valuable experience and references for other countries and regions worldwide on how to protect the mental health of youth groups during similar public health events (Chavira et al., 2022; Liu et al., 2024; Wang et al., 2022).

### Role of emotion regulation strategies

Stress associated with confinement has been linked to exacerbated negative cognitive biases, potentially impeding the cultivation of positive emotions (Somma et al., 2021). Psychological resources provide a strong buffering effect when individuals encounter stress events (Hu et al., 2025). Based on Gross's (1998) process model of emotion regulation, antecedent-oriented strategies like cognitive reappraisal enhance an individual's wellbeing. They achieve this by reinterpreting emotional stimuli prior to the full onset of emotional arousal. In contrast, response-oriented strategies such as emotional suppression are believed to interfere with the process of emotional processing. Moreover, they have the potential to intensify physiological stress (Gross and John, 2003; Webb et al., 2012). Emotion regulation, a well-studied coping strategy, is associated with blunted emotional response (Gross and John, 2003). Cognitive reappraisal and expressive suppression are two main strategies involved in cognitively interpreting the cause and consequence of emotional events (Gross, 2002).

During pandemic confinement, college students frequently encounter academic interruptions, social restrictions, and impacts of the pandemic itself (Pandya and Lodha, 2022). Cognitive reappraisal is expected to alleviate negative emotions through positive reinterpretation of stressors, while expressive suppression is associated with emotional exhaustion due to long-term suppression (Webb et al., 2012). Patrono et al. (2024)'s study on Chilean college students also showed that individuals with high emotion regulation ability had significantly lower somatization symptoms under strict confinement than those with low regulation ability, and the difference widened, reflecting the dynamic association between individual psychological resources and environmental restrictions. Individuals in long-term stressful environments exhibit obvious negative attentional bias, which may gradually deteriorate in confined environments; however, no current research has explained the underlying mechanism.

### Moderating role of attentional bias

Individuals also rely on trait-like mechanisms like attentional control ability and emotion regulation strategies when responding to stressful events (Olatunji et al., 2010). Skinner et al. (2023a) found that parental support and adolescents' own emotion regulation strategies, such as cognitive reappraisal, were crucial in buffering the impact of pandemic-related stress on internalizing symptoms in young adults across nine countries. Chronic stressors, exemplified by COVID-19 lockdown measures, deplete cognitive resources according to Folkman and Lazarus (2013) transactional theory of stress. This depletion can reshape the efficacy of emotion regulation strategies, notably increasing expressive suppression and self-compassion under prolonged stress (Liu et al., 2024). These findings highlight the importance of both individual and contextual factors in shaping mental wellbeing during the pandemic. Given its hypothesized interaction with emotion regulation strategies and key role in untangling pandemic—era mental health complexities, this study adopts attentional bias as the moderating variable.

### Current study

While numerous studies have investigated the pandemic's impact on mental health, gaps remain, particularly concerning the moderating role of confinement strategies and individual factors. Epidemiological surveys, while minimizing sampling bias, often overlook the influence of varying medical resources and residential density. To address this, our study focuses on universities of comparable level within the same city, employing different confinement strategies to examine their specific association with college students' mental wellbeing, as well as associated risk and protective factors. Specifically, we investigate the role of emotion regulation strategies (cognitive reappraisal and expressive suppression) and attentional bias (positive and negative) in mental wellbeing under varying confinement measurement. Furthermore, we aim to explore how the confinement state itself moderates the relation between these emotion regulation strategies and mental wellbeing. This research is grounded in Gross's (1998) process model of emotion regulation, Folkman and Lazarus (2013) transactional theory of stress, and Hobfoll's (1989) resource conservation theory, which collectively suggest that confinement, as a chronic stressor leading to resource depletion, may differentially influence the effectiveness of emotion regulation strategies, potentially making reappraisal a more adaptive strategy than suppression under such conditions (Wang et al., 2014). Given the mental wellbeing vulnerabilities of young adults during the pandemic, this study specifically examines Chinese college students' mental wellbeing during the COVID-19 pandemic.

Among Chinese college students, the confinement measures during the COVID-19 pandemic, as significant stressors, can influence the relation between emotion regulation strategies and mental wellbeing. Meanwhile, attentional bias, as an individual's tendency to process emotional information, can interact with emotion regulation strategies and further relate to mental wellbeing. Thus, we have selected emotion regulation strategies, mental wellbeing, confinement measures, and attentional bias as key variables to explore the relations among them, aiming to provide theoretical bases and practical guidance for improving the mental health of college students. Guided by the theoretical underpinnings discussed above and considering the unique context of Chinese college students during the pandemic, we propose the following hypotheses to systematically investigate the intricate relations among the key variables.

H1: Cognitive reappraisal positively promotes psychological wellbeing, whereas emotional expression suppression negatively impacts it. Cognitive reappraisal is associated with psychological wellbeing by altering individuals' cognitive evaluations of events. In contrast, emotional expression suppression is associated with the accumulation and repression of negative emotions, which are linked to adverse relations with mental wellbeing.
H2: Attentional biases moderate the impact of emotional regulation strategies on psychological wellbeing. Positive attentional bias may potentiate the positive influence of cognitive reappraisal, whereas negative attentional bias may exacerbate the negative impact of emotional expression suppression.
H3: Confinement measures moderate the impact of emotional regulation strategies on psychological wellbeing. Confinement measures may augment the positive influence of cognitive reappraisal while intensifying the negative impact of emotional expression suppression.

## Participants and procedure

This study was undertaken in Guangzhou, encompassing two universities to examine the differences between confined and unconfined situation during COVID-19 (April 15 to April 30, 2022). Among the two selected universities, one entered a confinement phase following the detection of a confirmed COVID-19 case near the campus in mid-April. Measurements were conducted approximately 10 days into this confinement period. Under this regime, students were restricted to the campus premises and prohibited from participating in indoor gatherings of more than ten people, while being allowed to engage in on-campus activities while strictly maintaining social distancing. In contrast, the other university remained unconfined throughout the study period, enabling students to live and study as usual. They could dine at on-campus cafeterias, attend classes in person, and leave the campus upon obtaining proper approval when necessary.

Thirteen thousand one hundred and nine valid questionnaires were collected in this study, and the age range of the subjects was 18–24 years old (20.28 ± 1.518 years). Among them, 5,513 (42.1%) were male subjects and 7,596 (57.9%) were female subjects. Five thousand two hundred and forty six subjects (40%) were from the confined situation and 7,863 subjects (60%) were from the unconfined situation.

All participants were screened to ensure they had no visual impairments, no history of neurological or psychiatric disorders, and were not currently using medication or recreational drugs, as self-reported through the online survey. Informed consent, tailored for both confined and unconfined settings, was meticulously obtained via the online survey platform, with each questionnaire initiated only after the participant's signature signified their understanding and agreement. Notably, while participants residing in the confined situation were not directly notified by the principal investigators about the specifics of confinement measures, electronic versions of the informed consent forms were widely disseminated through the online channel. This ensured that all participants had ample opportunity to thoroughly review and comprehend the content before submitting their data. Furthermore, the principal investigators remained accessible via telephone to address any queries or provide clarifications regarding the study's details, fostering a transparent and supportive research environment.

To ensure broad participation, researchers prepared an online invitation message containing their contact information and the link to an online questionnaire on the platform Wenjuanxing (www.wjx.com) indicating that completing the questionnaire would take approximately 10 to 30 min. This invitation was forwarded by university counselors to class monitors, who then shared it in class WeChat groups for college students. Participation was voluntary, and no remuneration was provided. Any questionnaire that was not fully completed due to premature withdrawal was deemed invalid and excluded from the database. Participants, after providing their informed consent, completed the questionnaire online, adhering to rigorous ethical protocols approved by the local ethics committee in alignment with the Helsinki Declaration's standards.

## Measures

### The emotion regulation questionnaire

The emotion regulation strategy scale used in this study was the Emotion Regulation Questionnaire (ERQ) developed by Gross and John (2003), translated and revised by Wang et al. (2007). The questionnaire consists of 10 items divided into two subscales: cognitive reappraisal (e.g., I control my emotions by changing the way I think about the situation I'm in) and expressive suppression (e.g., I keep my emotions to myself). A 7-point scale was used (1 = “strongly disagree” to 7 = “strongly agree”), with higher scores on a subscale indicating more use of the emotion regulation strategy by the subject. In this study, both the cognitive reappraisal subscale (Cronbach's α = 0.951) and the expressive suppression subscale (*Cronbach's* α = 0.854) had high reliability.

### The attention to positive and negative information scale

In this study, the Attention to Positive and Negative Information Scale (APNI) developed by Noguchi et al. (2006), revised and translated by Lv et al. (2016) was used to assess the extent to which individuals pay attention to positive and negative information in their. The scale consists of 30 items divided into two subscales: positive attentional bias (e.g., I mostly remember times when I was happy) and negative attentional bias (e.g., I can't forget the times I have performed poorly at something). A 5-point scale was used (1 = “strongly disagree” to 5 = “strongly agree”), with higher scores on the subscales implying that the subject's attentional bias was more toward that dimension. In the present study, both the positive attentional bias subscale (*Cronbach's* α = 0.972) and the negative attentional bias subscale (*Cronbach's* α = 0.972) had high reliability.

### The short Warwick Edinburgh Mental Well-Being Scale

In this study, the short Warwick Edinburgh Mental Well-Being Scale (SWEMWBS) was used to assess the mental wellbeing of individuals (Stewart-Brown et al., 2009). The scale consists of 7 items (e.g., I've been feeling optimistic about the future) and is scored on a 5-point scale (1 = “never” to 5 = “always”), with higher scores on the scale indicating better mental wellbeing. In the present study, *Cronbach's* α for the SWEMWBS was 0.960.

### Statistical analysis

The study used IBM SPSS 27.0 for data analysis. First, the common method bias was tested; second, the study used independent samples non-parametric tests to determine the mental wellbeing scores in areas with different confinement; again, to test the correlation between the variables, the study used Pearson correlation test to verify the significance of the correlation between variables; finally, to test whether negative/positive attentional bias moderate the relationships among variables, the study used PROCESS (model 1) to perform a moderating effect test. Any incomplete data was excluded from the database and not included in the subsequent analysis.

### Ethics approval statement

All procedures performed in studies that involved human participants were in accordance with the ethical standards of the institutional research committee (Ethics Commission of Tsinghua University, 2022 Ethics Review No. 19) and with the 1964 Helsinki declaration and its later amendments or comparable ethical standards.

## Results

### Common method bias control and testing

To assess the presence of common method bias (CMB), Harman's single-factor test was employed. Notably, the first factor accounted for 44.320% of the total variance, which is considerably lower than the commonly accepted threshold of 50%. This finding indicates that common method bias is not a significant issue in the current study.

### Correlation analysis

The correlation analysis among the variables is presented in Table 1. Cognitive reappraisal was strongly correlated with expression suppression and positive attentional bias and moderately correlated with negative attentional bias and mental wellbeing. Expression suppression was moderately correlated with positive attentional bias, negative attentional bias, and mental wellbeing. Positive attentional bias was correlated with negative attentional bias. Negative attentional bias was weak but significantly correlated with mental wellbeing. These findings indicate interrelated patterns among emotion regulation strategies, attentional biases, and mental wellbeing.

**Table 1:** Correlation analysis of variables.

**Variable**	**1**	**2**	**3**	**4**	**5**
1. Positive attentional bias	–				
2. Negative attentional bias	0.532^**^	–			
3. Cognitive reappraisal	0.743^**^	0.383^**^	–		
4. Expression suppression	0.508^**^	0.513^**^	0.730^**^	–	
5. Mental wellbeing	0.447^**^	0.112^**^	0.456^**^	0.316^**^	–

^**^p < 0.01.

### Normality test

In the normality test, when the *p*-value (i.e., significance) of the Kolmogorov-Smirnov (K-S) test is extremely small, such as 0.000, it indicates that there is sufficient evidence to suggest a significant difference between the data and the normal distribution, leading to the conclusion that the data does not follow a normal distribution. As shown in Table 2, the significance levels of Cognitive Reappraisal, Expression Suppression, Positive Attentional Bias, Negative Attentional Bias, and Mental Wellbeing are all 0.000, suggesting that none of them follow a normal distribution. Therefore, the Mann-Whitney U test was selected to analyze the differences in X_1_, X_2_, M_1_, M_2_, and Y between the confined and unconfined areas.

**Table 2:** Normality test table.

**Variable**	**Situation**	**Kolmogorov-Smirnova** ^**a**^		
		**Statistic**	**df**	**Significance**
Cognitive reappraisal	Unconfined situations	0.230	7,863	0.000
	Confined situations	0.207	5,246	0.000
Expression suppression	Unconfined situations	0.223	7,863	0.000
	Confined situations	0.234	5,246	0.000
Positive attentional bias	Unconfined situations	0.163	7,863	0.000
	Confined situations	0.129	5,246	0.000
Negative attentional bias	Unconfined situations	0.139	7,863	0.000
	Confined situations	0.132	5,246	0.000
Mental wellbeing	Unconfined situations	0.147	7,863	0.000
	Confined situations	0.171	5,246	0.000

^a^ Lilliefors significance correction.

### Differences in emotion regulation strategies, attentional biases, and mental wellbeing among college students between confined and unconfined areas

We employed non-parametric tests, specifically the Mann–Whitney U test and the Wilcoxon rank-sum test. The differential analysis revealed significant differences in cognitive reappraisal, expression suppression, positive and negative attentional biases, and mental wellbeing between confined and unconfined regions. Specifically, cognitive reappraisal, expression suppression, positive attentional bias, negative attentional bias, and mental wellbeing all showed substantial variations, indicating that confinement significantly impacts these psychological variables, see Supplementary Table S1.

To further investigate the association between confinement and cognitive reappraisal, expression suppression, attentional bias, and mental wellbeing, boxplots were generated for positive attentional bias, negative attentional bias variable across the two conditions.

**Figure 1:**
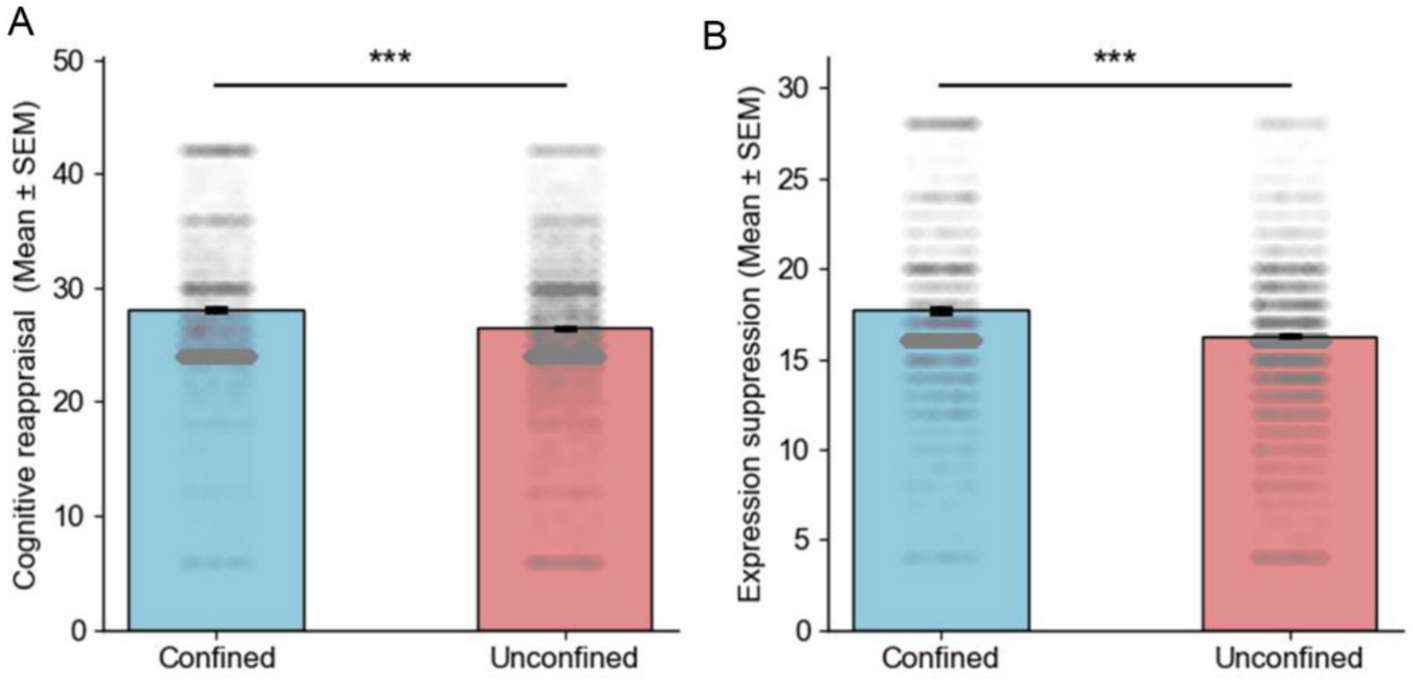
Bar graphs of Cognitive Reappraisal and Expressive Suppression in Confined and Unconfined Situation. **(A, B)** Bar graphs depict the mean values of cognitive reappraisal scores and expression suppression scores in confined and unconfined situations with enhanced emotion regulation strategies in confined situations (*** *P* < 0.001).

The results revealed that, in confinement, levels of cognitive reappraisal and expression suppression were higher compared to the general condition, with more dispersed data distributions and significant individual differences (see Figure 1). In contrast, in the unconfined condition, data distributions were more concentrated, and individual differences were relatively smaller.

**Figure 2:**
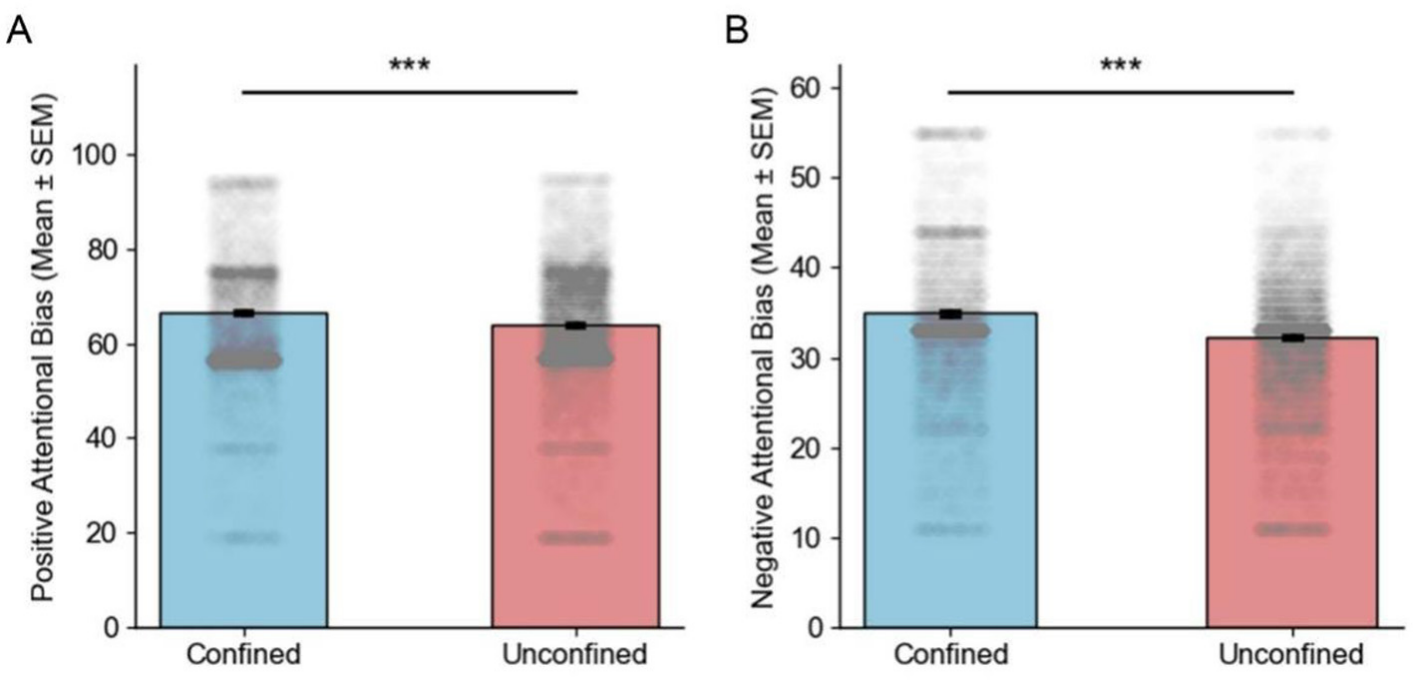
Bar graphs of attentional bias in confined and unconfined situation. **(A, B)** Bar graphs depict the mean values of positive attentional bias scores and negative attentional bias scores in confined and unconfined situations with enhanced both attentional bias in confined situations (*** *p* < 0.001).

Overall, both positive and negative attentional biases were higher under confinement compared to the general condition. In the confined situation, the data distribution was more dispersed, with greater individual differences dispersed data distributions and significant individual differences (see Figure 2). In contrast, in the non-confined situation, individual differences were relatively smaller, and the data distribution was more concentrated. The middle 50% of the data for negative attentional bias was distributed at a higher level, but with a large degree of dispersion and significant individual differences.

**Figure 3:**
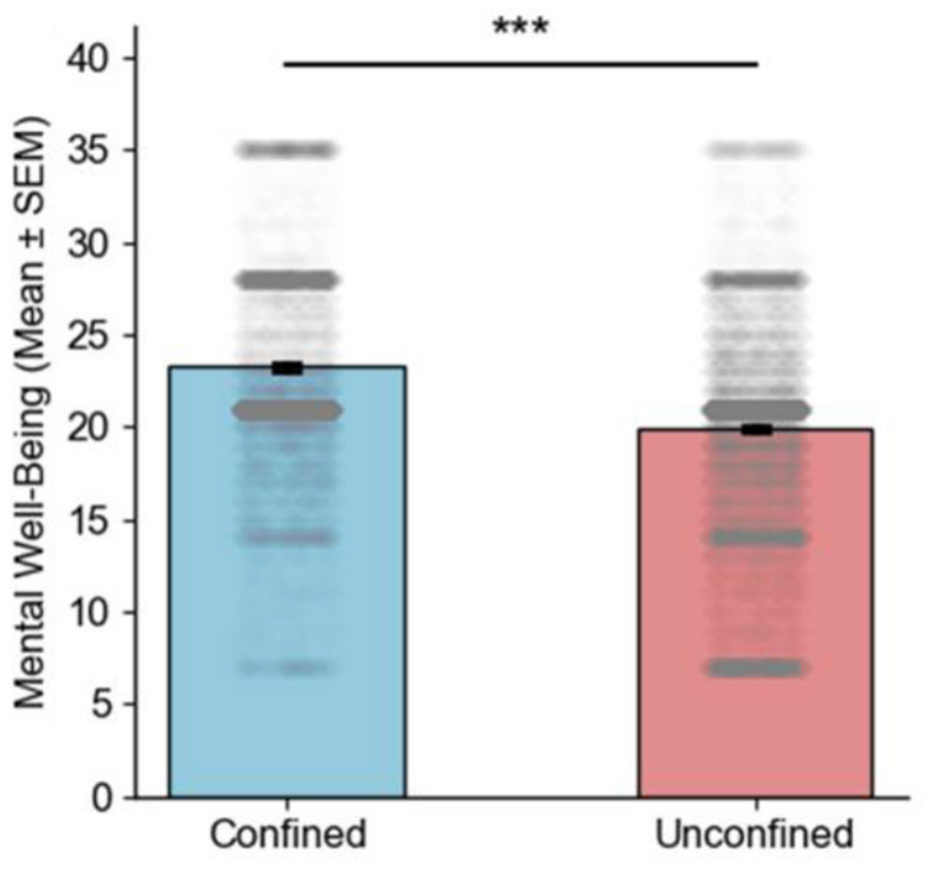
Bar graphs of mental well-being in confined and unconfined situation (***P < 0.001).

Mental wellbeing levels were generally higher under confinement, with the middle 50% of the data distributed at a higher-level distributions and significant individual differences (see Figure 3). However, the data dispersion was greater, indicating significant individual differences. In contrast, mental wellbeing data were more concentrated in the non-confined situation, with less dispersion, but overall levels were relatively lower compared to the confined condition. Additionally, more individuals exhibited lower levels of mental wellbeing in the non-confined situation relative to those under confinement.

### Moderation model with three independent variables

In this study, we examine the moderation model with two independent variables, where two independent variables, cognitive reappraisal and expression suppression, are moderated by three moderator variables, positive attentional bias, negative attentional bias and confined situation. Model 1 examined the direct effects of emotion regulation strategies (cognitive reappraisal and expressive suppression) on mental wellbeing. Model 2 incorporated positive/negative attentional bias as moderators, while Model 3 further included confinement status as a contextual moderator.

#### Initial models with single moderators

**Figure 4:**
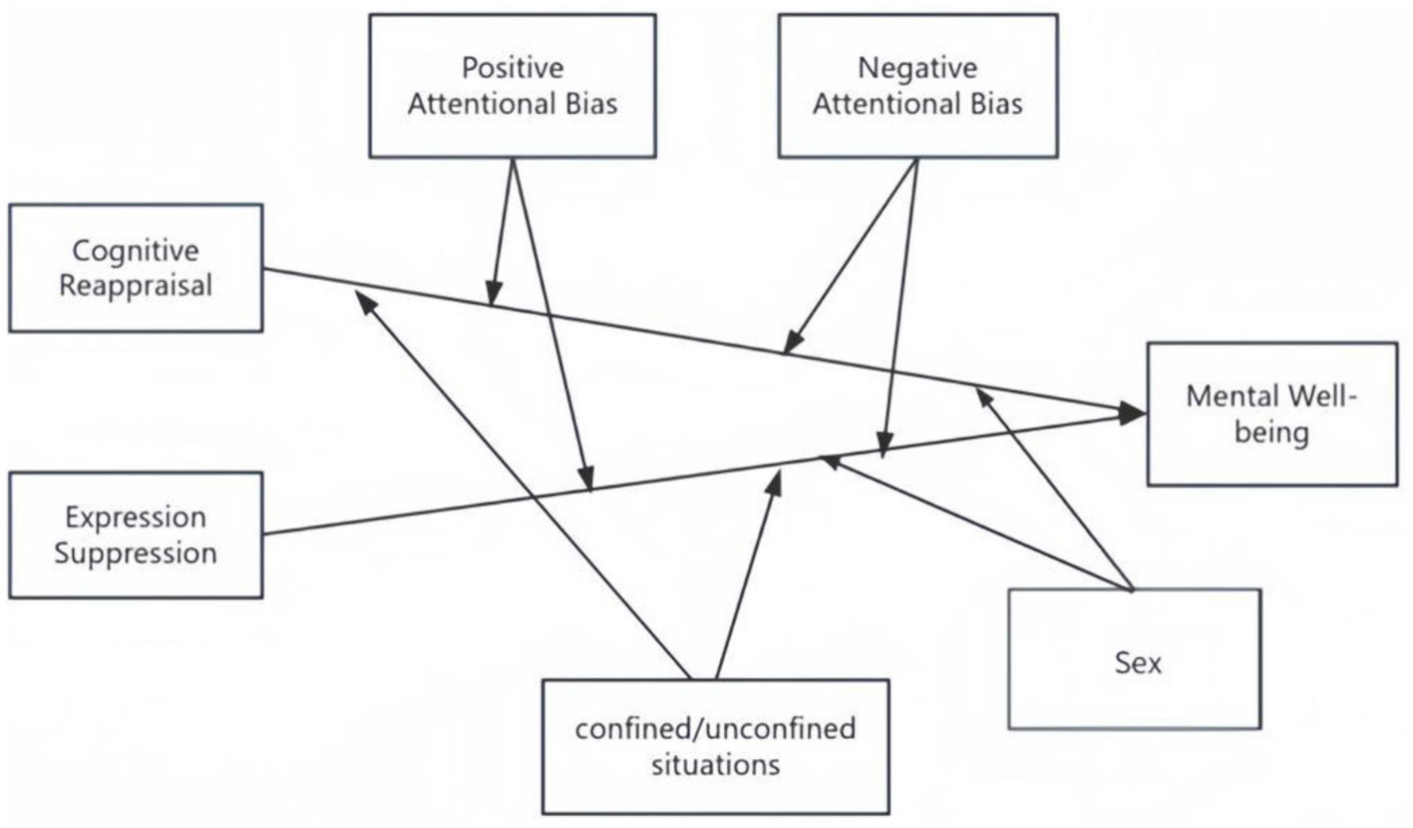
Moderation model with three independent variables.

In the initial analysis, we examined the effects of cognitive reappraisal (X_1_) and expressive suppression (X_2_) on mental wellbeing, with positive attentional bias (W_1_), negative attentional bias (W_2_), and regional differences (W_3_) each considered as moderators. Positive attentional bias had a significant positive effect on mental wellbeing (Beta = 0.114, *p* < 0.001) and enhanced the positive impact of expressive suppression (X_2_W_1_: Beta = 0.173, *p*=0.042), suggesting its role as a protective factor against psychological distress. Negative attentional bias had a significant negative effect on mental wellbeing (Beta = −0.186, *p* < 0.001), amplifying the negative impact of expressive suppression (X_2_W_2_: Beta = 0.519, *p* < 0.001) while attenuating the positive effect of cognitive reappraisal (X_1_W_2_: Beta = −0.286, *p* < 0.001). Regional differences also had a significant positive effect on mental wellbeing (Beta = 0.228, *p* < 0.001) and interacted significantly with both cognitive reappraisal (X_1_M: Beta = −0.170, *p*=0.001) and expressive suppression (X_2_M: Beta = 0.148, *p*=0.004), indicating that regional factors may influence the effectiveness of cognitive and emotional regulation strategies on mental well-being (see Figure 4). These findings provide context for subsequent model selection.

Based on the model presented in Table 3, the following regression equation can be formulated:


(1) $$\begin{array}{l} Y=b_{0}\;+\;b_{1}X_{1}\;+\;b_{2}X_{2}\;+\;b_{3}W_{1}\;+\;b_{4}W_{2}\;+\;b_{5}W_{3}\\ \;+\;b_{6}X_{1}W_{1}\;+\;b_{7}X_{1}W_{2}\;+\;b_{8}X_{1}W_{3}\;+\;b_{9}X_{2}W_{1}\\ \;+b_{10}X_{2}W_{2}\;+\;b_{11}X_{2}W_{3}\;+\;\epsilon \end{array}$$

**Table 3:** Moderation model with three independent variables.

**Variable**	**Model1**			**Model2**			**Model3**		
	**Beta**	**t**	**p**	**Beta**	**t**	**p**	**Beta**	**t**	**p**
Constant		39.733	0.000		33.074	0.000		21.590	0.000
Cognitive reappraisal (X_1_)	0.481	42.337	0.000	0.189	13.064	0.000	0.209	2.910	0.004
Expression suppression (X_2_)	−0.035	−3.095	0.002	0.069	5.836	0.000	−0.075	−1.049	0.294
Positive attentional bias (W_1_)				0.373	30.314	0.000	0.490	13.745	0.000
Negative attentional bias (W_2_)				−0.229	−23.888	0.000	−0.497	−13.020	0.000
Confinement (W_3_)				0.220	29.645	0.000	0.264	7.437	0.000
X_1_W_1_							−0.157	−1.773	0.076
X_1_W_2_							0.184	2.650	0.008
X_1_W_3_							−0.211	−4.156	0.000
X_2_W_1_							−0.045	−0.486	0.627
X_2_W_2_							0.255	3.653	0.000
X_2_W_3_							0.162	3.224	0.001
R_2_	0.208			0.305			0.311		
F	1,723.156			1,149.891			536.847		

Dependent variable: mental wellbeing.

Where:

*b*_0_ is the constant term.

*b*_1_ and *b*_2_ represent the direct effects of *X*_1_ and *X*_2_ on *Y*, respectively.

*b*_3_, *b*_4_ and *b*_5_ represent the direct effects of *W*_1_, *W*_2_, and *W*_3_ on *Y*, respectively.

*b*_6_ to *b*_11_ represent the moderating effects of the interaction terms on the relation between *X*_1_, *X*_2_, and *Y*.

ϵ is the error term.

When W_2_ and W_3_ are held constant, an increase of one unit in W_1_ results in a decrease of 0.157 units in the effect of X_1_ on Y and a decrease of 0.045 units in the effect of X_2_ on Y. These effects are not statistically significant.

When W_1_ and W_3_ are held constant, an increase in W_2_ enhances the positive effects of both X_1_ and X_2_ on Y by 0.184 and 0.255 units, respectively.

When W_1_ and W_2_ are held constant, an increase in W_3_ (confinement) weakens the positive effect of X_1_ on Y by 0.211 units but strengthens the positive effect of X_2_ on Y by 0.162 units.

Cognitive reappraisal (X_1_) was significantly positively associated with mental wellbeing in both Model 1 (Beta = 0.481, *t* = 42.337, *p* < 0.001) and Model 2 (Beta = 0.189, *t* = 13.064, *p* < 0.001). The moderation between cognitive reappraisal and positive attentional bias (X_1_W_1_) was not significant (Beta = −0.157, *t* =−1.773, *p*=0.076). The interaction between cognitive reappraisal and positive attentional bias was not significant (Beta = −0.157, *t* = −1.773, p = 0.076), but the interaction with negative attentional bias was significant (Beta = 0.184, *t* = 2.650, *p* = 0.008), suggesting that the relation between cognitive reappraisal and mental health is stronger at higher levels of negative attentional bias. Additionally, the moderation between cognitive reappraisal and regional coding (X_1_W_3_) was significant (Beta = −0.211, *t* = −4.156, *p* < 0.001), suggesting that the positive association between cognitive reappraisal and mental wellbeing was weaker in confinement compared to general zone.

Expression suppression (X_2_) was significantly negatively associated with mental wellbeing in Model 1 (Beta = −0.035, *t* =−3.095, *p* = 0.002) and significantly positively associated in Model 2 (Beta = 0.069, *t* = 5.836, *p* < 0.001). The moderation between expression suppression and negative attentional bias (X_2_W_2_) was significant (Beta = 0.255, *t* = 3.653, *p* < 0.001), indicating that higher levels of negative attentional bias were linked to a stronger negative association between expression suppression and mental wellbeing. The interaction between expression suppression and positive attentional bias (X_2_W_1_) was not significant (Beta = −0.045, *t* = −0.486, *p*=0.627), suggesting that positive attentional bias did not significantly moderate the relation between expression suppression and mental wellbeing. Additionally, the moderation between expression suppression and regional coding (X_2_W_3_) was significant (Beta = 0.162, *t*= 3.224, *p*=0.001), indicating that the association between expression suppression and mental wellbeing was stronger in confinement compared to general zone.

Positive attentional bias had a significant positive effect on mental wellbeing (Beta = 0.373, *t*= 30.314, *p* < 0.001), while negative attentional bias had a significant negative effect (Beta = −0.229, *t*= −23.888, *p* < 0.001). Regional coding (0–1) also had a significant effect on mental wellbeing (Beta = 0.220, *t*= 29.645, *p* < 0.001), with individuals in confinement showing higher levels of mental wellbeing compared to those in general zone.

The R-squared values indicated that Model 3 explained 31.1% of the variance in mental wellbeing (F = 536.847, *p* < 0.001), suggesting that the models provided a good fit to the data.

Building on the aforementioned models, we attempted to incorporate second-order interaction terms (W_1_W_2_, W_1_W_3_, and W_2_W_3_) and third-order interaction terms (X_1_W_1_W_2_, X_1_W_1_W_3_, X_1_W_2_W_3_, X_2_W_1_W_2_, X_2_W_1_W_3_, and X_2_W_2_W_3_) to further explore the moderating effects. However, these additional terms did not yield significant results and did not significantly improve the *R2* value thus these higher-order interaction terms were excluded from the model (Blumer et al., 1987). Consequently, we retained the original model as the most parsimonious and effective representation of the data.

#### Negative attentional bias moderation effect

**Figure 5:**
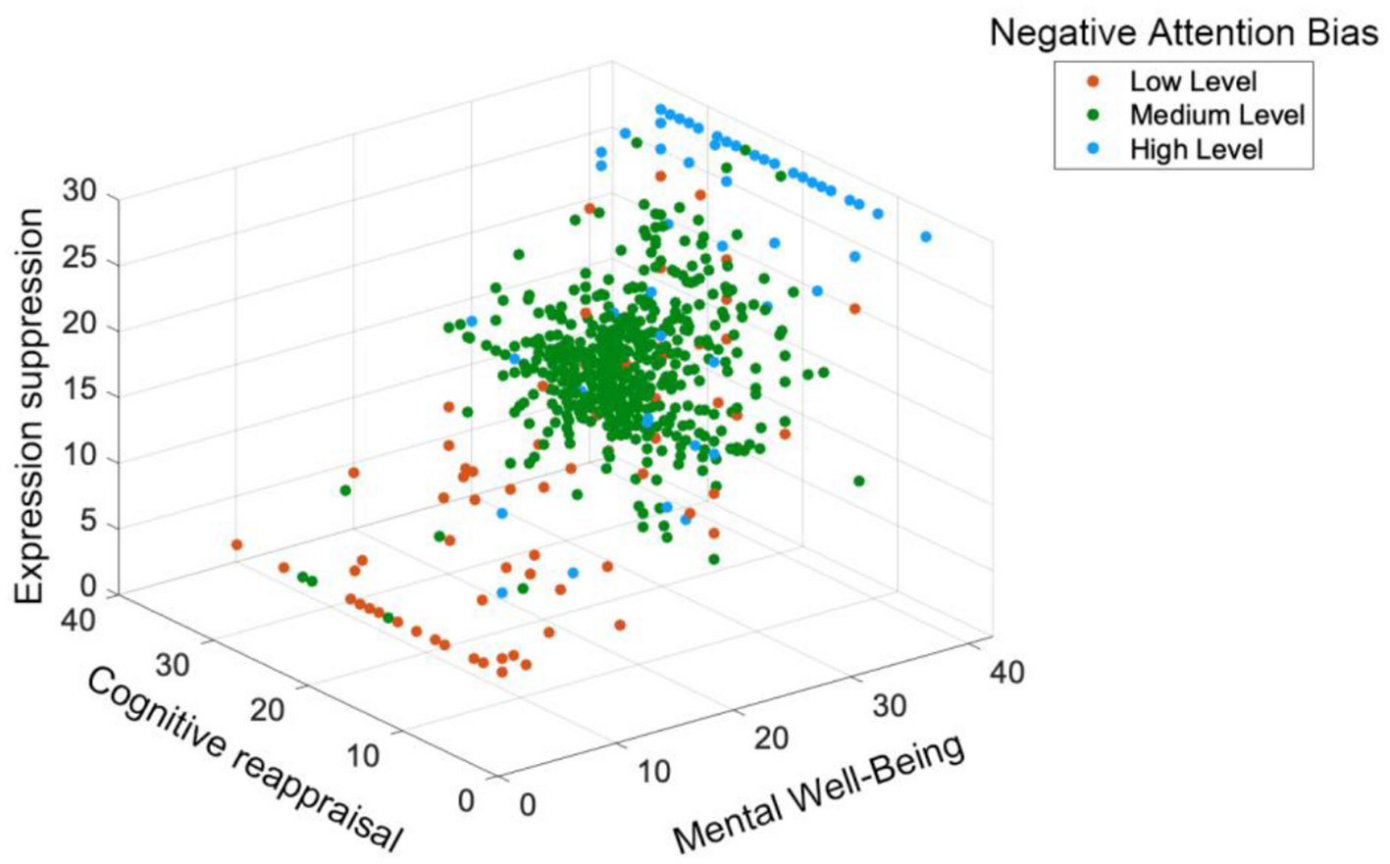
Three-Dimensional scatter plot of the moderating effect of negative attentional bias.

We constructed the three-dimensional moderation effect plot of negative attention using SPSS 27.0 (see Figure 5). For negative attention, the minimum value (11), the rounded mean value (33), and the maximum value (55) were selected as the representative values for the low, moderate, and high levels, respectively.

The red dots are primarily concentrated in the regions characterized by low cognitive reappraisal (0–30) and low expressive suppression (0–15). Within this region, mental wellbeing values are relatively low and widely dispersed. This suggests that when negative attention is at a low level, low cognitive reappraisal and low expressive suppression are often associated with poorer mental wellbeing, with significant individual differences.

The green dots are evenly distributed throughout the three-dimensional space, covering the entire range of cognitive reappraisal and expressive suppression values. In the regions with low cognitive reappraisal (0–20) and low expressive suppression (0–10), there is some overlap between green and red dots. However, the mental wellbeing values of green dots are relatively higher and more concentrated, indicating that when negative attention is at a moderate level, individuals may have better mental wellbeing even with low cognitive reappraisal and expressive suppression. As cognitive reappraisal and expressive suppression increase, green dots become more densely distributed in regions with higher mental wellbeing values, particularly in the regions with high cognitive reappraisal (30–50) and moderate expressive suppression (10–20). The increase in green dots in these regions suggests that under moderate levels of negative attention, higher cognitive reappraisal and moderate expressive suppression are more conducive to mental wellbeing, with a more pronounced positive effect.

The blue dots are mainly concentrated in the regions with high cognitive reappraisal (30–50) and high expressive suppression (20–30), where mental wellbeing values are relatively high and concentrated. This indicates that when negative attention is at a high level, high cognitive reappraisal and high expressive suppression are closely associated with better mental wellbeing.

Overall, the plot reveals that the moderator variable, negative attention, plays a significant role in modulating the relation between cognitive reappraisal, expressive suppression, and mental wellbeing. When negative attention is low, low cognitive reappraisal and expressive suppression are associated with poorer mental wellbeing, and improvements in these variables have limited effects on enhancing mental wellbeing. When negative attention is moderate, individuals tend to have relatively better mental wellbeing across various levels of cognitive reappraisal and expressive suppression, with higher cognitive reappraisal and moderate expressive suppression being particularly beneficial for mental wellbeing improvement. When negative attention is high, high cognitive reappraisal and high expressive suppression are closely linked to higher levels of mental wellbeing.

#### Confinement moderation effect

**Figure 6:**
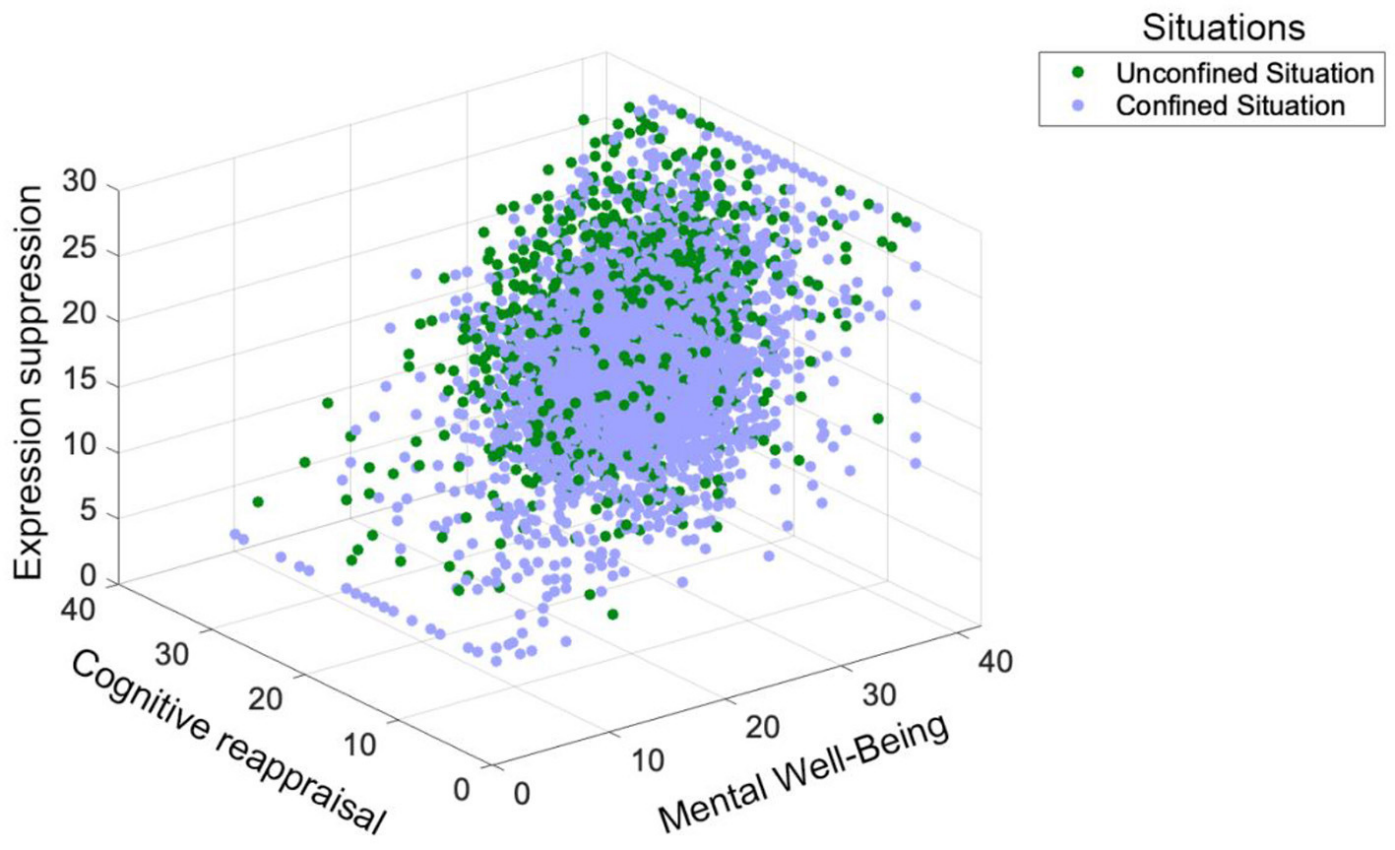
Three-dimensional scatter plot of the moderating effect of confinement.

We constructed the three-dimensional moderation effect plot of confinement using SPSS 27.0 (see Figure 6). The plot indicates that the situational variable (i.e., confined vs. unconfined situations) significantly influences the relation between cognitive reappraisal, expressive suppression, and mental wellbeing. Individuals in unconfined situations exhibit more diverse patterns across these variables, lacking distinct regional characteristics or regularities. In contrast, individuals in confined situations appear to have higher levels of mental wellbeing when engaging in higher cognitive reappraisal and moderate expressive suppression.

The green dots are widely distributed throughout the three-dimensional space, covering the entire range of cognitive reappraisal and expressive suppression values, with relatively uniform density across different regions. This suggests that individuals in unconfined situations display greater variability in cognitive reappraisal, expressive suppression, and mental wellbeing, without a clear concentration trend or specific pattern.

The distribution of blue dots overlaps with the green dots to some extent but also shows distinct differences. In regions with low cognitive reappraisal (0–20) and low expressive suppression (0–10), blue dots are relatively sparse. Conversely, in regions with high cognitive reappraisal (30–50) and moderate expressive suppression (10–20), blue dots are more densely concentrated. Moreover, mental wellbeing values in these regions are relatively higher and more concentrated. This pattern suggests that individuals in confined situations are more likely to achieve higher levels of mental wellbeing when they engage in higher cognitive reappraisal and moderate expressive suppression.

## Discussion

The current findings are largely consistent with the theoretical framework and hypotheses proposed in our study. The results confirmed that cognitive reappraisal exerts a positive influence on mental wellbeing, while expressive suppression demonstrates a negative association, which aligns with Gross's emotion regulation theory (Gross and John, 2003) and the assumptions of our H1. Similarly, the moderating roles of attentional biases and confinement measures in shaping the relation between emotion regulation strategies and mental wellbeing provide strong support for our H2 and H3, shedding light on the complex interplay between individual differences and contextual factors during the pandemic (Folkman, 2013; Hobfoll, 1989). Specifically, negative attentional bias was found to be associated with a stronger positive association between cognitive reappraisal and also strengthen the negative association between expressive suppression and mental wellbeing. Additionally, contextual differences, particularly confinement, play a crucial regulatory role. Specifically, confinement further enhances the positive impact of cognitive reappraisal while intensifying the negative association of expressive suppression with mental wellbeing.

The current study elucidated the impact of emotion regulation strategies on mental wellbeing, as well as their underlying moderating mechanisms. Cognitive reappraisal exerts a positive influence on mental wellbeing, yet this association is attenuated by confinement, and is enhanced by negative attentional bias. The impact of expression suppression on mental wellbeing is more complex, showing a shift from negative to positive effects across different models and is significantly moderated by negative attentional bias and confinement. Overall, the study underscores the significant role of attentional biases and confinement in shaping the relation between emotion regulation strategies and mental wellbeing, highlighting the necessity of considering the interplay between individual differences and situational contexts when promoting mental wellbeing.

### The impact of cognitive reappraisal and emotional expression suppression on psychological wellbeing

Cognitive reappraisal exerts a significant positive impact on psychological wellbeing, indicating that individuals who effectively engage in cognitive reappraisal tend to have better mental wellbeing. This association is consistent across different models, highlighting cognitive reappraisal as a robust protective factor for psychological wellbeing. As an antecedent strategy that occurs before emotions are fully activated, cognitive reappraisal efficiently guides emotional trajectories (Gross and John, 2003; Wang et al., 2021). The role of cognitive reappraisal in enhancing psychological wellbeing has been confirmed (Garnefski et al., 2001; Riepenhausen et al., 2022), and our results are in line with these previous findings, further validating this positive association in the context of our study on Chinese college students during COVID-19. Previous research (Fino et al., 2021a; Xu et al., 2020) has highlighted the effectiveness of cognitive reappraisal in buffering anxiety and promoting psychological growth under conditions of extreme stress. In addition, other evidence further supports that the habitual use of cognitive reappraisal contributes to better mental health and greater psychological resilience in the face of stressful situations (Cardi et al., 2021). Complementary evidence from medical students further corroborates this mechanism of cognitive reappraisal, showing that emotion regulation partially mediates the anxiety-depression link (Colonnello et al., 2022). Cross-cultural data reveal gender disparities: females reported heightened distress but concurrently demonstrated stronger social support and growth potential in both Italian (Fino et al., 2021b) and Albanian samples (Fino et al., 2022). Age-related vulnerabilities emerged particularly among young adults (18–24) and older adults (55–65), who exhibited elevated anxiety and avoidance coping during confinement (Fino et al., 2022).

Emotional expression suppression typically has a negative impact on psychological wellbeing, although this association is moderated by other variables such as attentional bias and contextual differences. Previous studies have found that expression suppression can exacerbate negative emotions and inhibit positive emotions, posing a threat to long-term wellbeing (Dryman and Heimberg, 2018; Kaplan et al., 2024). Its negative correlation with indicators of psychological wellbeing and positive correlation with adverse psychological states highlight the detrimental effects of expression suppression on wellbeing, life satisfaction, and depressive symptoms (Haga et al., 2009). However, findings from some researchers suggest that in collectivist settings, expressive suppression, a reactive regulation strategy, reduces emotional expression and is associated with better mental wellbeing (Tamir et al., 2024). Consistent findings from Tyra et al. (2021) indicate that there are no significant differences in the impact of expression suppression on mental wellbeing.

### The impact of attentional bias on mental wellbeing

Individuals with higher levels of negative attentional bias—a tendency to disproportionately focus on negative information—may restructure their concepts of negative stimuli, further promote seeking social support, reduce stress, and transform negative emotions into positive experiences, thereby enhancing mental wellbeing (Cardi et al., 2021). In contrast, positive attentional bias promotes the processing of positive information, leading to improved emotional states (Noguchi et al., 2006), life satisfaction (Riepenhausen et al., 2022), and overall mental wellbeing (Yeung et al., 2015). These findings are consistent with our observations in the current study.

Conversely, negative attentional bias can impair mental wellbeing by contributing to anxiety and depression (Liu et al., 2021), which is also reflected in our research results, showing that higher levels of negative attentional bias are associated with lower mental wellbeing scores in our sample. Emotional balance is crucial, as extreme biases can be detrimental (Grant and Schwartz, 2011). The Behavioral Immune System theory posits that humans have evolved defense mechanisms against pandemic threats, suggesting that heightened reactions to negative information are adaptive (Ackerman et al., 2018). Specifically, when individuals encounter aversive stimuli that may pose a disease threat, it elicits feelings of disgust as a protective mechanism (Makhanova and Shepherd, 2020). This mechanism may be related to the complex effects of negative attentional bias on mental wellbeing we found in our study, potentially influencing how individuals respond to the stressors during the COVID-19 pandemic.

### Moderating role of attentional bias

Negative attentional bias significantly influences the relation between cognitive reappraisal, emotional expression suppression, and psychological wellbeing. Negative attentional bias amplifies the negative impact of emotional expression suppression on psychological wellbeing and strengthens the positive effect of cognitive reappraisal. In contrast, positive attentional bias does not significantly modulate these relations.

Consistent with researchers (Aldao, 2013), cognitive reappraisal benefits individuals with negative biases by redirecting attention away from negative stimuli and toward positive/neutral stimuli. The link between expression suppression and negative attentional bias may stem from enhanced attentional control, redistribution of cognitive resources, and potentially heightened internal regulatory effort, thereby facilitating the processing of negative information. Processing negative and threatening information typically consumes more cognitive resources, and the psychological consequences of focusing on negative information are far greater than those of focusing on positive information. This is because negative information is evolutionarily more significant for our survival (Cianfanelli et al., 2023), prompting our attentional system to be inherently more inclined to focus on it.

### Impact of confinement on mental wellbeing

Reviews show the COVID-19 pandemic was associated with negative mental health effects across all ages. Pietrzak and Hanke (2024) analyzed 35 studies (2020–2023) in Poland, Spain, and the U.S., finding the pandemic was significantly associated with higher anxiety and depression levels globally, with stronger associations observed in younger individuals and females. Filindassi et al. (2022) reviewed 294 articles (2019–2020) and reported the first wave was linked to increased anxiety, stress, depression, and reduced wellbeing, with these associations being more pronounced among young people. As young adults, college students demonstrated significant mental health associations with the pandemic: Pandya and Lodha (2022) noted the pandemic was broadly correlated with anxiety, depression, and stress in college students, with some showing associations between pre-existing mental illnesses and symptom exacerbation.

Studies on adults highlight pandemic-related long-term psychological associations. Sharma and Sharma (2024) analyzed 13 UK studies (2020) on adults aged ≥65, finding the pandemic was associated with higher anxiety and depression symptoms. Wondmeneh and Solomon (2024) reviewed 20 Ethiopian studies (2020–2024), linking the pandemic to increased post-pandemic comorbid mental disorder prevalence, with stronger associations in females, unemployed individuals, and those with low social support. Wickens et al. (2023) scoped 66 global studies (up to 2021), showing pre-existing depression/anxiety/stress disorders were associated with symptom exacerbation and higher psychological distress. Kumar and Bhatia (2023) noted school closures during confinement were linked to deprivation of safe peer support and teacher support in children and adolescents, correlated with loneliness, irritability, and future uncertainty.

Confinement measures show complex associations with mental health across ages. Boonroungrut et al. (2022) reviewed 2,055 global studies (2020–2021), linking confinement to disrupted social support and altered learning environments, which were associated with mental health impacts on students from K12 to university levels. Kumar and Bhatia (2023) noted global school closures and confinement (2020–2022) were correlated with anxiety, depression, loneliness, sleep issues, and future uncertainty in children and adolescents, with stronger associations in low/middle-income regions like India, Africa, Asia, and Latin America. Kauhanen et al. (2023) identified school closures and social isolation as key factors linked to deteriorated mental health in children/adolescents, e.g., German 7–17-year-olds showed lower quality of life, increased hyperactivity, and peer problems, with more significant impacts on low-income/immigrant families. Wickens et al. (2023) emphasized isolation measures might be associated with disrupted treatment for pre-existing mental disorders or loss of social support, correlated with exacerbated depression/anxiety, particularly affecting specific populations.

College students have been particularly prone to mental health issues during confinement. Lei et al. (2020) found that the prevalence of anxiety symptoms among college students rose to 41.7% during isolation, with depression symptoms reaching 34.5%, significantly higher than in non-confined groups. Pietrzak and Hanke (2024) pointed out that college students experienced significant increases in anxiety, depression, and loneliness during confinement, especially with prolonged isolation and lack of social support. Studies on Chinese college students (Cao et al., 2020) showed that students in strictly locked-down areas had depression scores 15% higher than those in loosely controlled areas. Boonroungrut et al. (2022) cited research indicating that isolation exacerbated depression, anxiety, and loneliness in adolescents and children, along with increased behavioral problems.

The mechanisms through which confinement affects mental health are diverse. A US review of K12 students from March–July 2020 (Oosterhoff et al., 2020) revealed that confinement led to the loss of emotional support networks at school, increasing loneliness and decreasing a sense of belonging. Study by Schaefer et al. (2020) found that the shift in learning methods during isolation (e.g., stress from autonomous learning) exacerbated the psychological burden on students. A systematic review by Adjepong et al.'s (2022) systematic review of 25 studies across sub-Saharan Africa (2000–2023) found that isolation exacerbated disruption of social and emotional support, separated students from campus resources, and increased loneliness and academic pressure. The uncertainty of exam postponements and evaluations triggered anxiety, while scarce local mental health service resources limited students' access to professional help, making psychological issues prone to accumulation (Chadi et al., 2022; Gureje and Alem, 2000). Although some students alleviated stress through family support or self-regulation, the overall impact remained predominantly negative (Admin, 2023; Julius et al., 2024).

The impact of isolation on individual mental health varies with individual differences and environmental factors. Individuals with stronger psychological resilience, adequate social support, or effective stress-coping strategies experienced fewer negative effects from isolation (Martin et al., 2025). Environmental differences also played a significant role, with studies showing that home isolation had significantly less negative impact on mental health than centralized isolation (e.g., hotel quarantine) (Mohamed and Yousef, 2021). Some student groups experienced enhanced psychological security during isolation due to increased family time and reduced campus social pressure (Rogers et al., 2021). Changes in family interaction patterns indirectly influenced adolescents' mental health: harmonious family relationships and increased parent-child time spent together could have positive effects; remote work allowed parents more opportunities to accompany their children, potentially reducing parent-child conflicts and benefiting adolescents' mental health (Gencer et al., 2024, p. 26).

During the pandemic, some individuals gained psychological benefits from isolation through proactive coping measures. For example, regular exercise, cultivating new hobbies, self-reflection, organizing affairs, helping others, and fulfilling social obligations all contributed to enhancing psychological resilience and emotional health (Sharma and Sharma, 2024). Some individuals reported positive isolation experiences, such as “having more family time and relaxation, being able to refocus on and cherish existing things,” even viewing isolation as a “relaxation period,” which promoted mental health (Martin et al., 2025). By adopting coping strategies like planning, maintaining a regular lifestyle, and self-care, some individuals effectively maintained their mental health (Martin et al., 2025). Additionally, the “sense of control over flexibility” from autonomous learning brought short-term positive psychological effects to some students (Huck and Zhang, 2021; Schaefer et al., 2020; Youth Truth, 2020).

Research has revealed temporal effects in the impact of isolation on mental health: depression and anxiety symptoms might increase initially but significantly decrease over time (e.g., at the end of isolation) (Martin et al., 2025). Some regions or groups exhibited short-term positive effects at the beginning of isolation, such as the “honeymoon effect”—altruism and social support from collective crisis response led to a short-term decline in suicide rates (Goto et al., 2022). Reduced social pressure and increased family time brought short-term psychological improvements, but long-term isolation generally had negative impacts on mental health, especially for young groups (Pietrzak and Hanke, 2024). An observational study of 70–80-year-old elderly individuals showed that those who maintained physical activity through family activities did not experience significant worsening of depression symptoms despite increased sedentary time at the beginning of isolation (Sharma and Sharma, 2024). Rothenberg et al. (2024) conducted a longitudinal study on adolescents from multiple countries, observing that the impact of COVID-19 on their wellbeing varied over time and across cultures. These findings align with our study's focus on Chinese college students during the pandemic, highlighting the importance of considering the dynamic nature of the pandemic's influence on mental health.

According to the box plot and moderation model, the direct effect of confinement on mental wellbeing is significant, revealing that the mental wellbeing level in confinement areas is higher than that in general areas. Major public health events bring significant uncertainty to individuals. Previous studies have shown that, in the context of public health emergencies, the psychological states of individuals in different regions may exhibit a “ripple effect,” whereby individuals closer to the epicenter of the event perceive higher risks and experience more intense negative emotions (Kasperson et al., 1988; Wen et al., 2020). However, some studies also indicate that individuals near the epicenter of an epidemic or disaster often show lower levels of anxiety (Wang et al., 2021; Xie et al., 2011). This finding contradicts the “ripple effect.” Based on the “psychological eye of the storm effect” (Stewart-Brown et al., 2009), we speculate that this observed phenomenon may be related to risk stimulus adaptation in confinement areas. Combining Bandura's social learning theory (Wiegman et al., 1991; Wiegman and Gutteling, 1995), we hypothesize that individuals in central areas alleviate information-induced panic through direct experience (Wen et al., 2020). This adaptation and direct experience results in better mental wellbeing among people in confinement areas, due to their awareness of risks and prevention and confinement, compared to individuals not involved in confinement within the same city.

### Moderating role of confinement

The main effect of confinement situations on psychological wellbeing is positive, potentially reflecting an enhancement of psychological wellbeing among individuals through self-reflection, solitude, and other means in confinement situations. Our results further contribute to the growing body of evidence highlighting distinct gender and age patterns in pandemic-related psychological outcomes, consistent with studies showing younger adults are vulnerable to negative mental health effects during the pandemic lockdown (Lansford et al., 2023; Skinner et al., 2021). This finding aligns with Wen et al. (2020), who observed higher resilience in regions directly affected by COVID-19 due to adaptive coping strategies. However, confinement situations affect the impacts of cognitive reappraisal and expression suppression on psychological wellbeing through their moderating effects. Specifically, confinement situations limit the external support for cognitive reappraisal, thereby weakening its positive influence on psychological wellbeing; simultaneously, confinement situations exacerbate individuals' feelings of loneliness and stress, intensifying the negative impact of expression suppression on psychological wellbeing. This moderation effect may be explained by Bandura's social learning theory (Wiegman et al., 1991), where direct exposure to risk enhances adaptive regulation strategies but amplifies maladaptive strategies under prolonged stress.

### Implications and limitations

Despite its contributions, this study also acknowledges several limitations. Firstly, although the validated model is grounded in existing research and theoretical frameworks, the use of questionnaire-based and cross-sectional methods inherently precludes causal inferences. Future research may employ longitudinal designs to examine the causal effects of emotion regulation strategies on mental wellbeing. However, Wen (2017) has noted that questionnaire-based methods can still validate causal relations if the proposed causal sequence is logically, conceptually, and theoretically reasonable, and key confounding variables are controlled. Secondly, the study focused solely on the moderating effects of positive and negative attentional biases; future research should incorporate variables closely related to mental wellbeing, such as personality traits, social support, and psychological resilience, to comprehensively unravel the relation model between emotion regulation strategies and mental wellbeing. Lastly, the self-report method used to assess participants' behaviors may be prone to response biases, including social desirability effects (Paulhus, 1984). To mitigate measurement errors, future studies could incorporate behavioral experiments in conjunction with self-report measures. As noted by Rothenberg et al. (2024), gender and age can significantly impact how adolescents experience the disruptions caused by COVID-19. Their research showed that different genders and age groups may have varying levels of exposure to pandemic-related stressors and different coping mechanisms. In our study of Chinese college students, although we focused on the 18–24 age group, future research could delve deeper into how gender—specific factors interact with emotion regulation strategies and mental wellbeing, similar to how Skinner et al. (2021) found gender-related differences in the adjustment of young adults and their mothers during the pandemic.

## Conclusion

The results indicate that cognitive reappraisal and expression suppression have significant effects on mental wellbeing, with notable interactions involving attentional biases and regional differences. Specifically, negative attentional bias strengthens the positive effect of cognitive reappraisal and the negative effect of expression suppression on mental wellbeing. Regional coding also moderates these relations, highlighting the importance of contextual factors in understanding the dynamics of cognitive and emotional processes on mental wellbeing.

## Data Availability

The datasets presented in this study can be found in online repositories. The names of the repository/repositories and accession number(s) can be found in the article/Supplementary material.
