# Supplementary material for: Emotion regulation strategies and mental wellbeing among Chinese college students during COVID-19: the moderating roles of confinement and attentional bias
Source: Front Psychol. 2025 Jun 13;16:1571275. doi: 10.3389/fpsyg.2025.1571275 (PMC12202356; doi:10.3389/fpsyg.2025.1571275)
Supplement: Supplementary file 1 [file Supplementary_file_1.docx]

**Supplementary Information**

#### **Table S1 Differential Analysis Statistics**

| **Variable** | **Mann-Whitney U** | **Wilcoxon W** | **Z** | **Asymptotic Significance (Two-tailed)** |
| --- | --- | --- | --- | --- |
| Cognitive Reappraisal | 18,546,555.000 | 49,463,871.000 | -10.098 | .000 |
| Expression Suppression | 17,196,775.500 | 48,114,091.500 | -16.814 | .000 |
| Positive Attentional Bias | 19,206,682.000 | 50,123,998.000 | -6.700 | .000 |
| Negative Attentional Bias | 17,072,345.500 | 47,989,661.500 | -16.855 | .000 |
| Mental Well-being | 14,751,846.500 | 45,669,162.500 | -28.013 | .000 |

Note: a. Grouping variable: Region 0-1 coding (0 = unconfined, 1 = confined)

**Table S2-A Bivariate Positive Attentional Bias Moderation Effect Table**

| Variable | Beta | t | p |
| --- | --- | --- | --- |
| Constant | 10.435 | 19.160 | 0.000 |
| Cognitive Reappraisal (X1) | 0.247 | 4.093 | 0.000 |
| Expressive Suppression (X2) | -0.128 | -2.128 | 0.033 |
| W1 | 0.114 | 5.595 | 0.000 |
| X1W1 | 0.084 | 0.923 | 0.356 |
| X2W1 | 0.173 | 2.034 | 0.042 |
| R^2^ | 0.238 | | |
| F | 816.280 | | |

Dependent variable: Mental Well-being.

**Table S2-B Bivariate Negative Attentional Bias Moderation Effect Table**

| Variable | Beta | t | p |
| --- | --- | --- | --- |
| Constant | 13.098 | 24.319 | 0.000 |
| Cognitive Reappraisal | 0.640 | 7.264 | 0.000 |
| Expressive Suppression | -0.286 | -7.321 | 0.000 |
| W2 | -0.186 | -8.615 | 0.000 |
| X1W2 | -0.286 | -4.282 | 0.000 |
| X2W2 | 0.519 | 7.615 | 0.000 |
| R^2^ | 0.217 | | |
| F | 727.685 | | |

Dependent variable: Mental Well-being.

**Table S2-C Bivariate Confinement Moderation Effect Table**

| Variable | Beta | t | p |
| --- | --- | --- | --- |
| Constant | 8.854 | 29.857 | 0.000 |
| Cognitive Reappraisal | 0.507 | 37.270 | 0.000 |
| Expressive Suppression | -0.093 | -6.577 | 0.000 |
| Zone | 0.228 | 6.502 | 0.000 |
| X1M | -0.170 | -3.224 | 0.001 |
| X2M | 0.148 | 2.887 | 0.004 |
| R^2^ | 0.250 | | |
| F | 874.810 | | |

Dependent variable: Mental Well-being.

**Table S3 Moderating Effects with Interaction Terms of Moderators**

|  | **Beta** | **t** | **p** |
| --- | --- | --- | --- |
| Constant |  | 1.099 | 0.272 |
| Cognitive Reappraisa | 0.361 | 2.174 | 0.030 |
| Expression Suppression | 0.289 | 1.739 | 0.082 |
| Positive Attentional Bias | 0.809 | 13.342 | 0.000 |
| Negative Attentional Bias | 0.135 | 1.851 | 0.064 |
| Confinement | 0.706 | 7.570 | 0.000 |
| X1W1 | -0.496 | -1.996 | 0.046 |
| X1W2 | -0.307 | -1.112 | 0.266 |
| X1W3 | -0.283 | -0.800 | 0.424 |
| X2W1 | -0.425 | -1.827 | 0.068 |
| X2W2 | -0.667 | -2.230 | 0.026 |
| X2W3 | -0.141 | -0.410 | 0.682 |
| W1W2 | -0.811 | -7.314 | 0.000 |
| W1W3 | -0.372 | -2.044 | 0.041 |
| W2W3 | -0.164 | -0.919 | 0.358 |
| X1W1W2 | 0.697 | 1.939 | 0.052 |
| X1W1W3 | 0.289 | 1.003 | 0.316 |
| X1W2W3 | -0.095 | -0.430 | 0.668 |
| X2W1W2 | 0.912 | 2.493 | 0.013 |
| X2W1W3 | -0.063 | -0.206 | 0.837 |
| X2W2W3 | 0.369 | 1.785 | 0.074 |
| R^2^ | 0.321 | | |
| F | 309.428 | | |

Dependent variable: Mental Well-being.
